# Role of *Trypanosoma cruzi Trans*-sialidase on the Escape from Host Immune Surveillance

**DOI:** 10.3389/fmicb.2016.00348

**Published:** 2016-03-23

**Authors:** Ana F. F. R. Nardy, Celio G. Freire-de-Lima, Ana R. Pérez, Alexandre Morrot

**Affiliations:** ^1^Institute of Microbiology, Federal University of Rio de Janeiro Rio de Janeiro, Brazil; ^2^Carlos Chagas Filho Institute of Biophysics, Federal University of Rio de Janeiro Rio de Janeiro, Brazil; ^3^Institute of Clinical and Experimental Immunology of Rosario, CONICET, National University of RosarioRosario, Argentina

**Keywords:** *Trypanosoma cruzi*, *trans*-sialidase, parasites, immune evasion, glycoimmunology

## Abstract

Chagas disease is caused by the flagellate protozoan *Trypanosoma cruzi*, affecting millions of people throughout Latin America. The parasite dampens host immune response causing modifications in diverse lymphoid compartments, including the thymus. *T. cruzi trans*-sialidase (TS) seems to play a fundamental role in such immunopathological events. This unusual enzyme catalyses the transference of sialic acid molecules from host glycoconjugates to acceptor molecules placed on the parasite surface. TS activity mediates several biological effects leading to the subversion of host immune system, hence favoring both parasite survival and the establishment of chronic infection. This review summarizes current findings on the roles of TS in the immune response during *T. cruzi* infection.

## Introduction

The protozoan parasite *Trypanosoma cruzi* (*T. cruzi*) is the causative agent of Chagas disease. During their life cycle, *T. cruzi* acquires different morphologies, alternating between an insect vector and the vertebrate host (Tarleton et al., 2007; Ribeiro et al., 2012). Vector-borne transmission occurs when infected bugs take a blood meal releasing simultaneously contaminated feces with infective metacyclic trypomastigotes near the site of the mammal bite wound (Tyler and Engman, 2001). Once inside the host, parasites differentiate to bloodstream trypomastigotes, which can infect host cells where they differentiate into intracellular amastigotes.

Besides vectorial transmission, other forms of infection exist, i.e., organ transplantation, blood transfusion as well as vertical and oral transmission. Chagas disease progresses from an initial acute phase characterized by a large number of circulating parasites and a broad range of symptoms (same individuals can develop fever, muscle pain, lymphadenopathy or an inflammatory reaction at the biting site known as chagoma), to a chronic and asymptomatic phase where the parasite load is nearly undetectable (Devera et al., 2003; Tarleton et al., 2007). Such latent stage could persist for the lifetime of individuals. However, nearly 30% of chronically infected individuals progress to a symptomatic disease, with the development of cardiomyopathy, megacolon, or megaesophagus (Coura and Borges-Pereira, 2010).

The parasite has evolved diverse mechanisms to subvert or escape from the host innate and adaptative immune system. One of them is the induction of an immunosuppressive state, which was described both during the acute phase of experimental and human *T. cruzi* infection (Oladiran and Belosevic, 2012).This condition is characterized by anergy or clonal deletion of T lymphocytes as well as polyclonal activation of B cells with production of low affinity antibodies against *T. cruzi* (Ortiz-Ortiz et al., 1980; Maleckar and Kierszenbaum, 1983). Thus, the inhibition of host immunity observed during the acute phase is an essential way for parasite persistence and the consequent establishment of chronic disease.

Sialic acids (SAcs) are a family of nine-carbon monosaccharides present on the surface of all mammalian cells, conferring diverse biologically activities to glycoproteins and glycolipids, like the promotion of cell–cell interactions or masking recognition sites due to its negative charge (Frasch, 2000). SAcs act as recognition receptors for diverse pathogens including viruses, bacteria and parasites (Varki, 1997; Esko and Sharon, 2009). Moreover, pathogenic bacteria like *Escherichia coli K1* and *N. meningitidis* synthesize SAcs and use it to decorate their surfaces to evade the immune system in their mammalian hosts (Vimr and Lichtensteiger, 2002). Unlike these microorganisms, *T. cruzi* is unable to synthesize SAcs *de novo*. Since *T. cruzi* requires SAcs to survive in the mammalian *milieu*, the parasite exploites the presence of SAcs on host cells, transferring the monosaccharide from host sialyl-glycoconjugates to terminal β-galactoses of acceptor molecules located on its own surface. This enzymatic activity is carried out by an unusual parasite enzyme known as *trans*-sialidase (TS), a modified sialidase (Previato et al., 1985; Freire-de-Lima et al., 2015).

TS displays a diversity of biological properties (many of them independent of their enzymatic activity), which promote the evasion of the innate and adaptative immune responses, acting as an important *T. cruzi* virulence factor (Burleigh and Andrews, 1995). The comprehension of mechanisms involving TS in the abrogation of immunity against *T. cruzi* infection is crucial for the developing and establishment of effective therapeutic approaches.

## *Trypanosoma cruzi Trans*-Sialidase: A Glance on its Characterization and Structure

The first description of the presence of SAcs residues on *T. cruzi* surface came from studies performed in the eighties (Pereira et al., 1980). Later, it was demonstrated that SAcs found on the parasite surface were previously transferred from the extracellular *milieu*, since no conventional precursors were found on parasites (Schauer et al., 1983). Furthermore, it was determined that trypomastigotes also displayed neuraminidase activity, because they hydrolyzed SAcs residues from erythrocytes and plasma glycoproteins (Pereira, 1983). Finally, Previato et al. (1985) demonstrated that *T. cruzi* performs the enzymatic transference of SAcs by an alternative route involving a *trans*-glycosylation reaction. Subsequent studies demonstrated that such *trans*-glycosylase activity is specific for alpha(α)2,3-SAcs (Schenkman et al., 1991). Further, genetic studies performed to characterize *T. cruzi*-TSs genes showed that *trans-*sialidase and neuraminidase activities were associated to the same parasite enzyme (Pereira et al., 1991; Parodi et al., 1992; Uemura et al., 1992).

*Trypanosoma cruzi*-TS is part of the TS-superfamily, encoded by 1430 genes. There have been characterized ∼15 genes for enzymatically active-TS, and more than 700 for the inactive-TS which lack the catalytic domain; while the rest are pseudogenes (Atwood et al., 2005; El-Sayed et al., 2005). Moreover, TS-superfamily was recently classified by genomic analysis in eight groups. Active-TS members belong to the group-I, while inactive-TS members belong to groups-II to VIII (Freitas et al., 2011).

**FIGURE 1:**
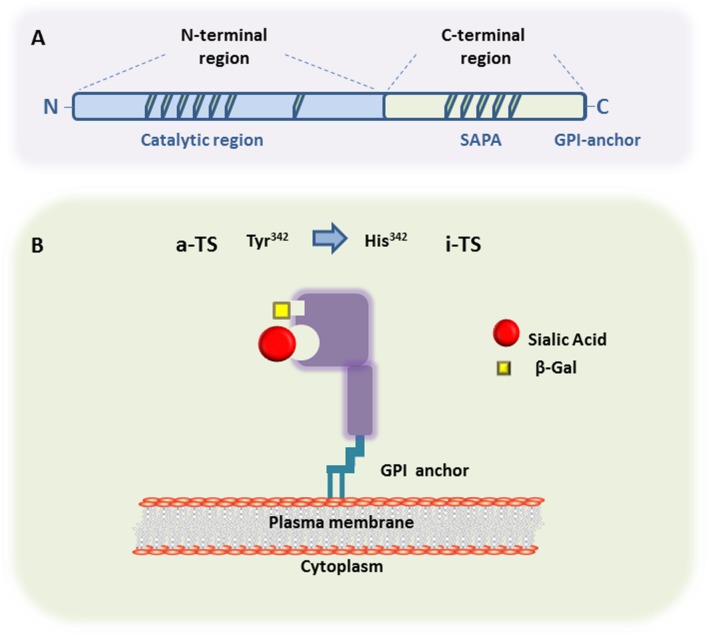
***Trans*-sialidase structure.** **(A)** Representative scheme of the primary structure of metacyclic- and a bloodstream trypomastigote-TS members belonging to either Group I (active-TS) or Group II (inactive-TS), showing the N-terminal and C-terminal domains. **(B)** Representative 3D structure of active (a-TS) and inactive (i-TS) forms of *Trypanosoma cruzi*-TS. *T. cruzi-*TS proteins are bound to the parasite surface through GPI-anchors. Active-TS sialylates parasite’s mucin-like molecules, as well as host cell surface glycoconjugates. Inactive-TS in turn acts as a parasite adhesin and it is differentiated from a-TS by a single mutation in the catalytic residue of Tyrosine at the position 342, which is commonly changed by a Histidine.

The general TS protein structure derived from metacyclic and bloodstream trypomastigote forms shows two major regions. As seen in **Figure 1**, one region is a N-terminal catalytic region whereas the other one consist of a C-terminal region displaying repeats of 12 amino acids in tandem, called SAPA (by Shed Acute Phase Antigen) (Pollevick et al., 1991). The hydrophobic catalytic pocket, responsible for the interaction of transferred SAcs with the terminal β-galactose acceptor, is a three-dimensional structure rich in aromatic residues (Buschiazzo et al., 2002). Inactive-TS members belonging to the TS group II (Freitas et al., 2011) differ from the active ones in their N-terminal region by the presence of mutations in catalytic residues causing the lack of enzymatic activity (Tyr^342^ by His^342^ is the commonest mutation) (**Figure 1B**). These inactive-TSs are lectin-like proteins since they maintain the capacity of binding SAcs and β-galactose residues (Cremona et al., 1995, 1999; Oppezzo et al., 2011), and are involved in host cell attachment and invasion (Freitas et al., 2011). Furthermore, both TSs are surface glycophosphatidylinositol (GPI)-anchored molecules, which can be released into the bloodstream by the action of phosphatidylinositol phospholipase C on GPI-anchors (Schenkman et al., 1992).

## The *Trypanosoma cruzi Trans*-Sialidase is Able to Overcome the First Line of the Immune Defenses

**FIGURE 2:**
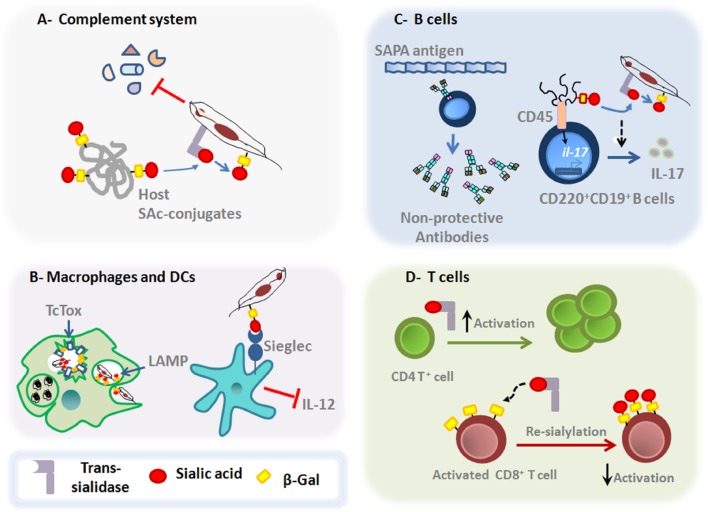
**Main roles of *T. cruzi* TS on the host immune responses.** **(A)** Upon entry in the host *milieu*, which is rich in SAcs donors, the parasite surface gets a negatively charged barrier due to a reaction catalyzed by TS, conferring protection against the complement-mediated killing. **(B)** TS favors *T. cruzi* escape from the parasitophorous vacuole, process in which LAMP and Tc-Tox proteins are also shown to be involved. Moreover, TS is able to interfere with IL-12 secretion by dendritic cells through the interaction between sialylated molecules on *T. cruzi* surface and Siglecs on dendritic cell surface. **(C)** SAPA-antigen induces the production of non-protective antibodies. Moreover, recent findings have show that TS acts on both mature follicular and marginal zone CD220^+^CD19^+^B cells to induce the expression of IL-17 by a non-canonical signaling pathway. These signaling events promote changes in the glycosylation profile of CD45 that result in their phosphatase activity and subsequent activation of downstream signals leading to the *Il-17* gene expression. **(D)** TS can also induce the activation of CD4^+^T cells *in vivo*. In addition, TS can also promote changes in the sialylation pattern of activated CD8 ^+^T cells, dampening the protective functions of MHC class I-dependent lymphocyte killing responses against *T. cruzi* parasites.

As sialic acid residues can be found on the surface of all mammalian cells, exerting crucial roles in regulating both innate and adaptative host immunity (Amon et al., 2014), it is not surprising that *T. cruzi* takes advantage of such host cell sialoglycophenotype. In this sense, in addition to transfering SAcs to the parasite surface, the TS can also transfer SAcs between host cell glycoconjugates, allowing the parasite to affect the host immune response (**Figure 2**).

The subversion of immune response by *T. cruzi* depends at first on their early action upon innate compounds. The host *milieu* is plenty of potential SAcs donors, allowing the parasite to acquire a negatively charged cover surface right after entering into the host, through the reaction catalyzed by TS. The fact of acquiring such negatively charged mask enables *T. cruzi* to circumvent the effects of some serum compounds (Vimr and Lichtensteiger, 2002). The elimination of this protective cover by sialidase treatment, make trypomastigotes more susceptible to the complement-mediated lysis (Kipnis et al., 1981). In addition, GPI-anchored surface GP160/CRP and T-DAF proteins, putative members of inactive-TS family, conferred protection against the complement-mediating killing by avoiding C3 convertase assembly (Tomlinson et al., 1994; Norris, 1998) (**Figure 2A**). Furthermore, sialylglycoconjugates present on the parasite surface due to TS activity, can bind SAcs binding Ig-type lectins (Siglec-E) on dendritic cells, suppressing the production of the proinflammatory cytokine IL-12, thus impairing the central branch connecting the innate and adaptative immune responses and favoring immunosuppression (Erdmann et al., 2009) (**Figure 2B**).

*Trypanosoma cruzi* can infect any nucleated cell, including phagocytic cells. To facilitate the adhesion and invasion of mammalian host cells, *T. cruzi* parasites use different molecules, including TS (Barrias et al., 2013). The reduced ability of *T. cruzi* to invade SAcs-deficient cells compared to wild type cells, clearly show that key steps of parasitism, such as the adhesion and invasion of host cells require the participation of TS (Ming et al., 1993; Schenkman et al., 1993). Moreover, both active- and inactive-TS seem to participate in these steps (Barrias et al., 2013). After cellular binding, *T. cruzi* invades non-phagocytic host cells by diverse processes that alternatively require fusion of lysosomes at the site of entry, participation of plasma membrane components or fusion with endosome compartments at the site of invasion (de Souza et al., 2010; Barrias et al., 2013). Regardless of the mechanisms of infection, the parasite will be later located in a vesicle called parasitophorous vacuole (Tardieux et al., 1992; de Souza et al., 2010). The internal membrane of this organelle is enriched with highly sialylated LAMP proteins (Albertti et al., 2010).The acidic pH of the parasitophorous vacuole favors the activity of TS in transferring SAcs from LAMP proteins to the parasite surface. The desialyzation process renders the vesicle more prone to lysis by the action of Tc-Tox (a parasite-derived protein with membrane pore-forming activity), enabling the parasite to escape into the cytoplasm (Andrews and Whitlow, 1989; Hall et al., 1992; Rubin-de-Celis et al., 2006; Albertti et al., 2010) (**Figure 2B**).

*Trypanosoma cruzi* parasites can be also internalized via phagocytosis by macrophages, a process that triggers an oxidative burst to kill parasites (Alvarez et al., 2011). However, the parasite can avoid macrophage-induced cytotoxicity using an antioxidant complex system to ensure their escape into the cytoplasm in order to establish a productive infection (Piacenza et al., 2009; Nagajyothi et al., 2012).

Moreover, TS can also remodel the surface of surrounding host cells. As mentioned earlier, TS can be shed into the bloodstream, causing removal of SAcs located on platelet surface, an event that increases their clearance thus favoring the occurrence of thrombocytopenia (Tribulatti et al., 2005).

## *Trypanosoma cruzi Trans*-Sialidase Compromises Both B and T Cell Responses

During the acute phase, it has been shown the involvement of TS in the polyclonal B-lymphocyte activation (Gao and Pereira, 2001). Probably, the B cell polyclonal activation may restrict the niche size required for an optimal development of specific and protective lymphocytes, by increasing the competition for activation and survival signals in the lymphoid organs (Freitas and Rocha, 2000; Montaudouin et al., 2013). Interestingly, the presence of immunodominant epitopes derived from shed acute phase antigens (SAPAs) shared by TS, drives the production of non-inhibitory antibodies against regions close to the catalytic site of the enzyme (Alvarez et al., 2001) (**Figure 2C**). These antibodies are produced in a T cell-independent manner, delaying the acquisition of inhibitory antibodies (Gao and Pereira, 2001; Gao et al., 2002). Importantly, these inhibitory antibodies are mainly neutralizing IgG immunoglobulins directed against the catalytic site of TS, and their production is strongly correlated with an IFN-γ enriched *milieu* (Pereira-Chioccola et al., 1994; Ribeirão et al., 2000; Risso et al., 2007). It is possible that the SAPA antigens have evolved to avoid the production of protective antibodies directed against the N-terminal catalytic region, since they are absent in the epimastigote forms. Additionally, these antigens are responsible to increase the half-life of TS shed into the bloodstream (Ribeirão et al., 1997; Buscaglia et al., 1999). Furthermore, the trypomastigote surface is rich in terminal α-galactosylmucins, which are also targeted by lytic antibodies. Nevertheless, the *T. cruzi* parasites can avoid the killing induced by human anti-α-galactosyl antibodies by the highly sialylated negatively charged surface (Chioccola et al., 2000; Buscaglia et al., 2006).

Interestingly, recent data demonstrate that during *T. cruzi* infection, B220^+^CD19^+^B cells can produce IL-17, a cytokine involved in the protective response against the parasite (ToselloBoari et al., 2012; Bermejo et al., 2013; Erdmann et al., 2013) (**Figure 2C**). Increased levels of IL-17 in the infection seems to be driven by active-TS acting on CD45 mucins located on B220^+^CD19^+^B cell surface, in a ROR-γt/AhR-independent manner (Bermejo et al., 2013). Despite that this type of B cells could be considered as innate cells, such results point out that B cells and different CD45 isoforms are also targets of *T. cruzi*-TS activity (Freire-de-Lima et al., 2010; Muia et al., 2010).

Differentiation, homeostasis and activation processes of T cells greatly depend on the sialylation process. Thus, *T. cruzi*-TS-mediated modifications of the T cell sialophenotype indeed influence the host immune response.

During acute phase of *T. cruzi* infection, T cells become anergic and undergo clonal deletion. Moreover, T cells exhibit low IL-2 expression and are more prone to suffer apoptosis after activation (Nunes et al., 1998; Alcaide and Fresno, 2004). Interestingly, the induction of CD4^+^Th1 and CD8^+^ cytotoxic T-cell protective responses is partly elicited by TS (Rodrigues et al., 1999). Both active- and inactive-TS forms engage the CD43 sialomucin in CD4^+^T cells, favoring their activation (**Figure 2D**). Such mechanism is shown to be able to rescue T cells from activation-induced cell death, intensifying the mitogenic capacity of these lymphocytes (Todeschini et al., 2002). Furthermore, the antigen-specific CD8^+^T cell responses are regulated by a distinct sialylation process (Moody et al., 2001; Pappu and Shrikant, 2004). During T cell activation, the expression of SAcs on lymphocyte surface is downregulated, leading to the exposure of desialylated glycans (Priatel et al., 2000). Such decreased sialylation process is important to augment the reactivity of T cells for their cognate peptide in the context of MHC class I molecules (Kao et al., 2005). In this context, recent studies showed that TS is able to re-sialylate the surface of CD8^+^T lymphocytes, limiting the acquisition of antigen-specific CD8^+^T cell responses (**Figure 2D**). These findings suggest that the re-sialylation ability of TS over activated T cells is an important parasite evasion strategy that directly influences the *T. cruzi* half-life time inside the host by preventing the immune control elicited by CD8^+^T cells (Freire-de-Lima et al., 2010). In fact, a large number of epitopes recognized by CD8^+^T cells in both experimental *T. cruzi* models and human disease is encoded by the TS family of genes, although the studies reveal that the epitope immunodominance of TS members varies according to the parasite strain (Martin et al., 2006).

## Role Of *Trypanosoma cruzi Trans*-Sialidase In The *T. cruzi-*Driven Thymic Atrophy and Development of Unconventional Extrathymic T Cell Subsets in Chagas Disease

The thymus is the primary lymphoid organ where bone marrow-derived T cell precursors undergo differentiation. As a result of this process, positively CD4^+^ and CD8^+^ selected thymocytes migrate as mature T cells to T-cell areas of peripheral lymphoid organs (Savino and Dardenne, 2000). Several infectious pathologies, including Chagas’ disease, promote disturbances of the intrathymic compartment (Savino, 2006). During the acute phase of murine *T. cruzi* infection is commonly observed an intense thymic atrophy, mainly caused by the depletion of immature CD4^+^CD8^+^ double-positive (DP) thymocytes. Such phenomenon involves not only thymocyte death but also their abnormal proliferation and migration responses (Leite de Moraes et al., 1991; Roggero et al., 2002; Henriques-Pons et al., 2004).

**FIGURE 3:**
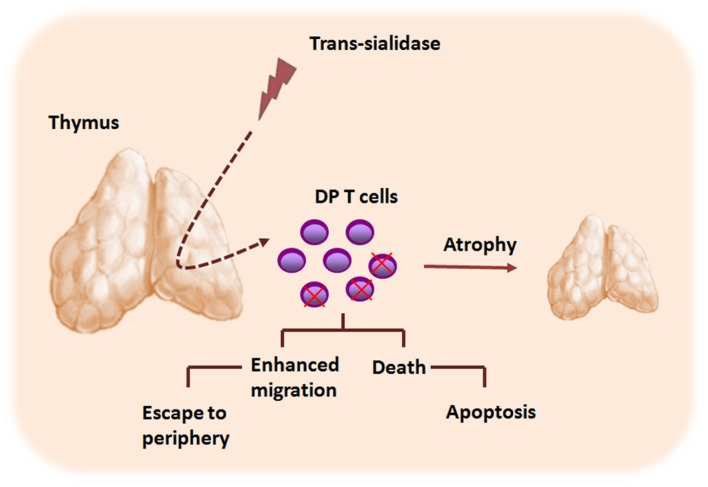
***Trypanosoma cruzi* TS contributes to the thymic atrophy seen during acute phase of Chagas disease.** The thymic atrophy caused by *T. cruzi* parasite is a complex phenomenon and involves several host–parasite molecule interactions, including those elicited by TS. TS promotes CD4^+^CD8^+^ double positive (DP) cells apoptosis within the thymic nurse cell complex, contributing to the thymic atrophy process. Moreover, TS also modulates the migratory properties of thymocytes, and may contribute to the abnormal release of DP thymocytes to the periphery.

Interestingly, changes in the cell surface sialylation caused by TS seem also to play an important role in *T. cruzi*-induced morphological and phenotypic thymic alterations (**Figure 3**). When TS is artificially shedding to circulation, the parasite enzyme is able to induce apoptosis of thymocytes. In addition, even when TS doses became undetectable, thymic apoptosis could also be observed (Leguizamón et al., 1999). Moreover, TS-treated animals displayed enhanced thymocyte apoptosis within the thymic nurse cell complexes, findings resembling thymic alterations in infected animals. Additionally, the TS treatment was able to promote a decrease of thymocyte proliferative ratios after Concanavalin A stimulation, in a similar manner to those observed in the experimental models of *T. cruzi* infection. The use of TS-neutralizing antibodies apparently rescued the normal thymocyte proliferation indexes (Mucci et al., 2002). Importantly, the thymocyte apoptosis induced by TS depends on androgens, since TS administration in both female and androgen-depleted mice did not result in the increase of thymocyte death. Furthermore, lactitol, a competitive inhibitor of TS that blocks the transference of sialyl residues by TS, was able to prevent the thymic cell depletion induced by *T. cruzi* (Mucci et al., 2005, 2006). Further studies showed that a rise in the glucocorticoid levels, as consequence of infective stress, is also involved in the *T. cruzi*-driven thymic atrophy (Roggero et al., 2006; Pérez et al., 2007; Lepletier et al., 2013). Interestingly, it has been reported that glucocorticoids can change the expression of sialylated and nonsialylated Lewis (a) epitopes of adhesion molecules involved in leukocyte migration processes (Delbrouck et al., 2002). Whether similar results occur during *T. cruzi*-driven thymic atrophy it remains to be determined.

Finally, recently studies from our group revealed that TS influences the thymocyte differentiation process via activation of MAPK signaling pathways, increasing thymocyte migratory activity by inducing actin filament mobilization. Such effects were also related to the ability of TS to modulate the adhesive properties of thymocytes to thymic epithelial cells. Moreover, we found increased frequencies of activated DPT cells in the blood of chronic chagasic patients with heart disease, in association to elevated titters of anti-TS antibodies as compared to healthy individuals (Nardy et al., 2013). These findings suggest a probably role for TS in the intrathymic maturation disturbances and subsequent abnormal thymic exit of immature thymocytes seen in *T. cruzi* infection.

## Concluding Remarks

A successful establishment of an infection relies on efficient strategies adopted by the infectious agents to evade early their detection by the host immune system. Considering that protozoans are the most ancient members of the animal kingdom, it is conceivable to think that they evolved sophisticated mechanisms to ensure their survival as intracellular parasites. *T. cruzi* is part a diverse group of unicellular organisms that can modify host cells to their own benefits (Jackson, 2015). In this review, we highlighted important aspects of *T. cruzi* immune evasion involving TS, a major parasite virulence factor. The diverse functions displayed by this molecule enable the parasite to interfere in many crucial host biological processes, including those responsible for protective immune responses. Despite several years of research directed toward understanding the role of *T. cruzi-*derived TS on the host-parasite interplay, there are still some points to be uncovered, especially those involving the distribution of genes encoding TS in different parasite strains. This question becomes even more important if we consider the coexistence of different *T. cruzi* strains in their natural reservoirs (Noireau et al., 2009). The existence of antigen variation within the parasite population, may lead the expression of competing T cell epitopes with different affinities to MHC molecules that could influence the acquisition of protective adaptive immune responses. Given the importance of TS for the establishment of an efficient infection, this molecule has gained potential attention as a drug target for disease therapies. Thus, efforts to understand the biology of TS would strengthen the use of TS inhibitors in therapeutic approaches for treatment of Chagas disease.

## Author Contributions

AN, CF, and AP wrote the paper. AM made substantial contributions to the conception of the work. All authors read and approved the final version of the manuscript.

## Conflict of Interest Statement

The authors declare that the research was conducted in the absence of any commercial or financial relationships that could be construed as a potential conflict of interest.
